# Administration of melatonin prior to modified synchronization protocol improves the productive and reproductive efficiency of Chinese crossbred buffaloes in low breeding season

**DOI:** 10.3389/fvets.2023.1118604

**Published:** 2023-05-12

**Authors:** Adili Abulaiti, Mudussar Nawaz, Zahid Naseer, Zulfiqar Ahmed, Wenju Liu, Mohamed Abdelrahman, Aftab Shaukat, Ahmed Sabek, Xunsheng Pang, Shujuan Wang

**Affiliations:** ^1^College of Animal Science, Anhui Science and Technology University, Fengyang, AnHui, China; ^2^Anhui Province Key Laboratory of Animal Nutritional Regulation and Health, Fengyang, AnHui, China; ^3^Faculty of Veterinary and Animal Sciences, Pir Mehr Ali Shah Arid Agriculture University, Rawalpindi, Pakistan; ^4^Key Laboratory of Swine Genetics and Breeding, Huazhong Agricultural University, Wuhan, China; ^5^Key Laboratory of Agricultural Animal Genetics, Breeding and Reproduction, Huazhong Agricultural University, Wuhan, China; ^6^College of Life and Health Science, Anhui Science and Technology University, Fengyang, AnHui, China; ^7^Department of Veterinary Hygiene and Management, Faculty of Veterinary Medicine, Benha University, Benha, Egypt

**Keywords:** melatonin, milk traits, fertility, synchronization, summer season, Chinese crossbred buffaloes

## Abstract

**Introduction:**

Melatonin is a neurohormone involving various biological processes, including restoration of cyclicity in animals with seasonal breeding patterns. The use of melatonin in different forms has gained broader acceptance in different species, particularly in summer anestrous buffaloes.

**Objectives:**

The objective of the current study was to evaluate the melatonin effect on the reproductive and productive performance of crossbred buffaloes during the low breeding season.

**Methods:**

Sixty-five cyclic and reproductively sound crossbred buffaloes were randomly allocated to three groups: the G1 (*n* = 20) served as the control group and received no single melatonin, G2 received melatonin (*n* = 22; 18 mg/50 kg, body weight) once prior to synchronization and G3 group was administered multiple melatonin injections (*n* = 23; 6 mg/50 kg body weight) for three consecutive days before the start of the synchronization protocol. The reproductive performance, milk yield traits, and serum immunoglobulin M (IgM) and melatonin levels were evaluated in treated and untreated crossbred buffaloes.

**Results:**

The results revealed that a single dose of melatonin administration has (*p* < 0.05) improved estrus response, ovulation occurrence and follicular growth in crossbred buffaloes compared to control groups. Higher pregnancy rates were observed in both melatonin-treated buffalo groups compared to the control. Following the administration of melatonin, serum IgM level increased in G2 and G3; however, an increment in melatonin level (*p* < 0.05) was detected in the G2 group only as compared to the control group subsequent day of melatonin administration. The milk compositions were not affected by melatonin administration except for milk urea nitrogen and somatic cell count (SCC). The melatonin administration (*p* < 0.05) decreased the somatic cell count in buffalo milk compared to untreated.

**Conclusion:**

In conclusion, single or multiple doses of melatonin before initiating the synchronization protocol improved the ovulation, ovulatory follicle diameter and pregnancy rates in crossbred buffaloes during the low breeding season. Moreover, the administration of melatonin enhanced the IgM values along milk traits in terms of milk protein, MUN and somatic cell count in treated buffaloes.

## 1. Introduction

The water domestic buffalo (*Bubalus bubalis*) has played an essential role in the agricultural economy of Asian countries (1). Buffalo is a source of milk, meat, and draft power in different countries. The water buffalo are categorized into two main types: the riverine and the swamp. The Murrah, Surti, Jafarabadi and Nilli-Ravi are the main river buffalo breeds in the Indian subcontinent (2), while Mediterranean breeds exist in Balkan and east Europe (3). The swamp breeds are mainly concentrated in Southeast Asia to the Yangtze valley of China (4). The buffaloes are continuous polyestrous, and ovarian activity and conception rates vary throughout the year. The resumption of ovarian activity occurs in shorter day-length periods (October–March) in tropical environmental conditions, and this period is also termed the “peak breeding period” in buffaloes (5). In contrast, the heat stress months decreased the duration and intensity of estrus and pregnancy rate in buffaloes when the temperature-humidity index (THI) exceeded>25 in the summer season (6–8) due to alteration in endocrine milieu (9, 10). Additionally, the summer climate has a noticeable effect on follicle size and number, oocyte developmental competence and blastocyst quality in buffaloes (11–13). Increased day length and hyperprolactinemia also resulted in the suppression of gonadotropin secretion and steroidogenesis (14). In addition to heat stress, the influence of seasonal breeding patterns on the reproductive activity of buffalo is a recognizable factor governed by melatonin release. Melatonin release occurs throughout the year, but their duration, according to the photoperiod, drives the gonadotropin and steroidogenesis, a seasonal breeding phenomenon in buffaloes.

Melatonin also acts as an antioxidant and lowers the incidence of apoptosis in ovaries, improving gamete and steroid production (15). The exogenous melatonin administration proves this phenomenon in buffaloes during the low breeding season (16, 17). Moreover, melatonin has been applied along estrus and ovulation synchronization in anestrous heifers and buffaloes to improve fertility in the summer season (18–22). Melatonin is involved in sexual maturation and resumption of ovarian functions by activating its receptors (MTNR1A and MTNR1B) and binding sites in the HPG hypothalamic–pituitary-gonadal (HPG) axis. In GnRH secretion, melatonin improves calcium ions influx to GnRH-expressing neurons and regulates the HPG axis by up-and down-regulation of gonadotropin gene expression in response to season (23). In addition, increased melatonin concentration in the follicle is a biomarker for improved steroidogenesis, follicular development, oocyte maturation and subsequent fertilization (24, 25).

In addition to the beneficial impact of melatonin on reproduction, exogenous melatonin is used as an immune enhancer, antioxidant, and somatotropin in different animal models (26–30). The long summer days affect milk production by controlling the interplay of prolactin and IGF-1 and antioxidant levels in buffaloes (31). The somatic cells count in milk is an indirect indicator to measure the udder health status of dairy animals (32). The increment in somatic cells indicates the diseased udder, which results in the decline of milk production and quality (33). Using exogenous melatonin injections is also a beneficial way to lower the somatic cell count in the milk of Holstein cows (27). Considering the seasonal aspects of breeding in buffaloes, immunity, reproductive and productive performance in response to exogenous melatonin have yet be elucidated in Chinese crossbred buffaloes. Therefore, it is necessary to observe the variations in melatonin and immunoglobulin levels following exogenous administration of single or multiple melatonin injections and, secondly, to study the milk traits and reproductive performance after synchronization of melatonin-treated Chinese crossbred buffaloes.

## 2. Materials and methods

### 2.1. Care and use of animals

Prior to the execution of experiments, approval (Approval ID: HZAUBU-2017-001) was obtained from the Animal Welfare and Ethical Committee, Huazhong Agriculture University, People’s Republic of China. All experimental procedures were conducted according to the proposed guidelines by the Animal Welfare and Ethical Committee.

### 2.2. Description of experimental animals and husbandry practices

The present study was conducted during the low breeding season (May to August) at a private buffalo farm (Hubei Jinniu Co. Ltd.) Hubei Province, China (latitude 30° 32′ N, longitude 111° 51′ E). The Chinese crossbred buffaloes (*n* = 65; Nili Ravi × Jiangha) were cyclic with normal calving history and sound reproductive tract. The selected buffaloes were multiparous (3–4 parity), possessing average body weight (588.12 ± 89.25 kg) and good BCS (2.5–3.5). Regular ultrasonography helped to exclude the acyclic (presence of corpus luteum) and uterine-infected buffaloes. Buffaloes remained in a good state of health throughout the experiment. Buffaloes had free and easy access to fresh and clean water and were fed a total mixed ration (TMR) two times daily. TMR was formulated according to NRC (2001) with 16.89% crude protein (CP, % DM) and 1.58 net energy (Nel, Mcal/kg DM).

The ambient temperature (°C) and relative humidity (RH) were recorded routinely. The temperature-humidity index (THI) was calculated according to the following formula (34):


$$\mathrm{THI}=\left(0.8{}^{\circ}T{}^{\circ}C\right)+(\mathrm{RH}/100\times \left(T{}^{\circ}C-14.4\right)+46.4$$

### 2.3. Experimental design of modified estrus synchronization protocols

**Figure 1:**
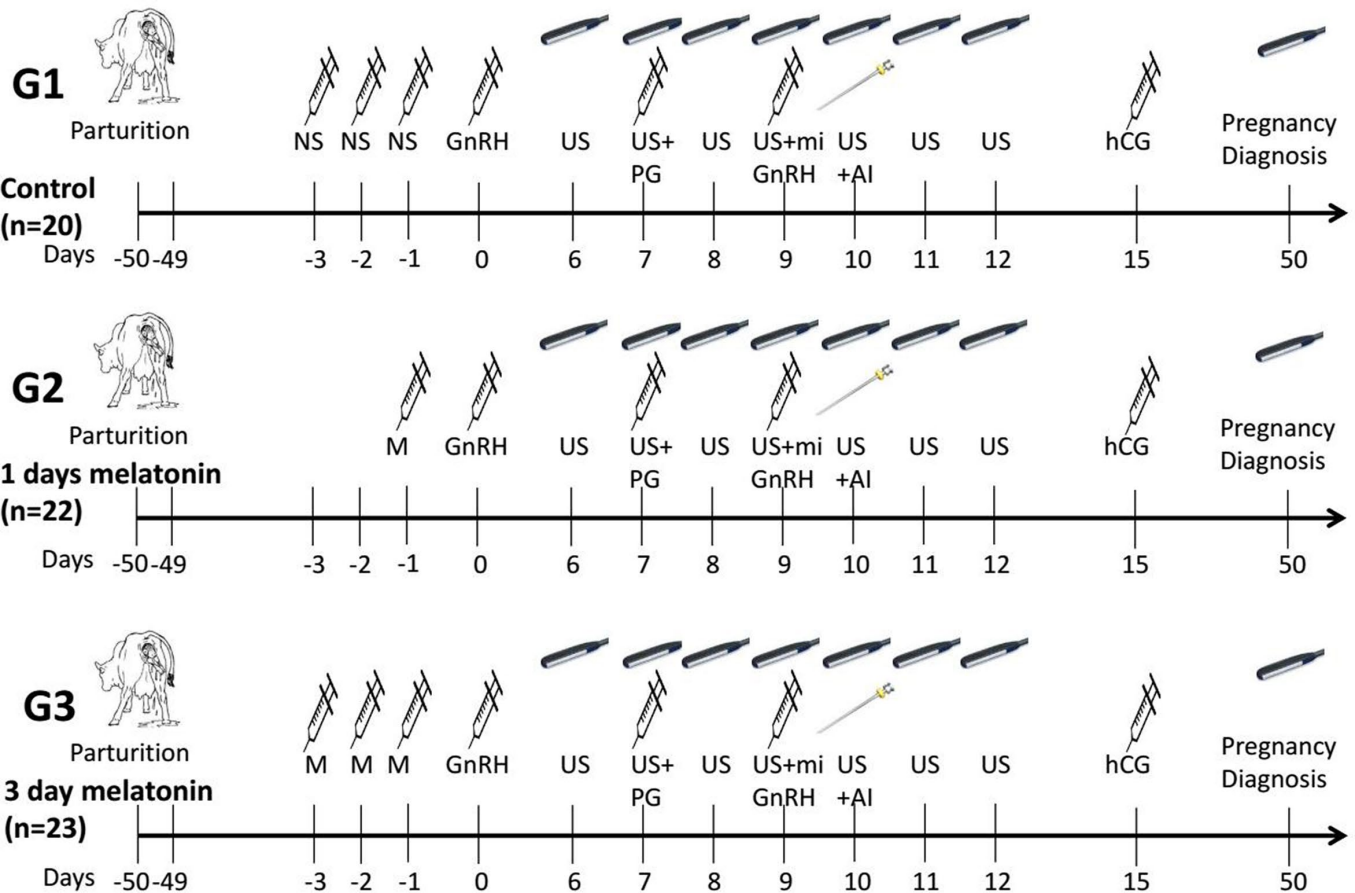
Schematic representation of the study design of modified ovsynch protocol in crossbreed buffaloes. NS, Normal saline; US, Ultrasonography; PG, Prostaglandin; GnRH, Gonadotropin releasing hormone; hCG, Human chorionic gonadotropin; M, Melatonin; mi, mifepristone; Al, Artificial insemination.

The schematic representation of the experimental design is given in Figure 1. The selected buffaloes were randomly allocated into three treatment groups based on melatonin dosages. The normal saline solution (6 ml/50 kg BW) was injected subcutaneously during three consecutive days (day −3, day −2 and day −1) before the start of the synchronization protocol in the control group (G1; *n* = 20). The melatonin (Vita-Pure Inc. Roselle, NJ, United States) was dissolved in 75% ethanol solution, and a final volume of 5 ml, containing 6 or 18 mg melatonin, was prepared by diluting it in physiological saline. The melatonin (18 mg/50 kg BW) was injected one time (day −1) subcutaneously to buffaloes of the G2 group (n = 22) a day prior to the start of the synchronization protocol. In comparison, a dose of melatonin (6 mg/50 kg BW) was injected subcutaneously for three consecutive days (day −3, day −2 and day −1) before the start of the synchronization protocol in the third group (G3; n = 23). The modified estrus synchronization protocol was started with an intramuscular (I.M.) injection of gonadotropin-releasing hormone (200 μg/head, day 0, Ningbo Sansheng Pharmaceutical Ltd., China) after 50 days of calving. The prostaglandin (PGF2α, 0.5 mg, I.M., Ningbo Sansheng Pharmaceutical Ltd., China) was injected on day 7 of the protocol. Then, a second dose of GnRH and mifepristone (0.4 mg/kg, I.M., Hubei Yun Cheng Sai Technology, China) was administered on day 9, and artificial insemination was performed on day 10. In addition, human chorionic gonadotropin (hCG, 2000 IU, I.M., Ningbo Sansheng Pharmaceutical Ltd., China) was injected intramuscularly on day 15 of the protocol. Pregnancy diagnosis was conducted on day 50 by ultrasonography.

### 2.4. Reproductive parameters

The follicular dynamics were observed by ovarian scanning using an ultrasound scanner (WED-9618-v, LV2-3/6.5 MHz rectal probe, Shenzhen Well.D. Medical Electronics Co. Ltd. Guangdong, China) from day 6 to day 12 (Figure 1). Ultrasonography was performed every 12 h to record the follicular dimensions. The sudden disappearance of the dominant follicle (>10 mm) after the second GnRH (day 9) was delineated as ovulation. The presence of a persistent follicle (>15 mm) on the last ovarian scan (day 12) was categorized as an anovulatory follicle. Buffalo estrus response was recorded by daily visual observation of estrus signs (edematous vulva, mucus discharge, and sniffing of genitalia). The conception rate was calculated by dividing the number of pregnant buffaloes by the number of artificially inseminated buffaloes.

### 2.5. Milk production and milk composition

Daily total milk yield was recorded in liters using calibrated jars after each milking time (06:00 h and 16:00 h). Milk composition traits, milk fat (%), milk protein (%), lactose (%), non-fat solids (%), total solids (%), milk urea nitrogen (mg/dL) and somatic cell count (10^3^/ml), were analyzed using mid-infrared spectroscopy a MilkoScan FT+ (Foss, Hillerød, Denmark) on day 5 and 10 after last melatonin injection.

### 2.6. Estimation of melatonin and IgM

Blood samples were collected during the dark hours (18:00 to 21:00) on different days (0, 1, 5, and 10 days) after melatonin injection to detect the concentration of melatonin and IgM in the G2 and G3 groups.

The melatonin level was measured in samples by the double antibody sandwich method. The absorbance (OD value) was measured with an enzyme marker at the wavelength of 450 nm, and the content of bovine melatonin in the sample was calculated through the standard curve. The melatonin level was estimated according to the procedure described manufacturer (Shanghai Enzyme-linked Biotechnology Co., Ltd.). The melatonin concentration was detected using bovine melatonin ELISA kit (CEA908Ge 96 T Enzyme-linked immunosorbent assay kits of Shanghai Enzyme Link Biotechnology Co., Ltd., China) with sensitivity 4.27 pg/ml and intra-assay variation <10%. Melatonin level was calculated by using the following formulae:

$$\begin{array}{c} \mathrm{OD}\;\mathrm{value of melatonin}=\mathrm{OD}\;\mathrm{value of measuring tube}\\ -\mathrm{OD}\;\mathrm{value of control tube} \end{array}$$

The concentration of melatonin (pg/ml) was calculated by the linear regression equation of the standard curve by using the standard concentration and OD value and substituting the OD value of the sample into the equation of the standard curve. After calculating the melatonin concentration in the sample, multiply by the dilution factor for the actual concentration of the sample.

The level of IgM in the samples was determined using the double antibody sandwich method mentioned above. The absorbance (OD value) was measured with an enzyme marker at the wavelength of 450 nm, and the standard curve calculated the content of IgM in the sample. IgM as an immunity indicator was estimated using Bovine Immunoglobulin M (IgM) ELISA Kit (KL-IgM-Ca 96 T Enzyme-linked immunosorbent assay kits of Shanghai Enzyme Link Biotechnology Co., Ltd., China) with the specification of sensitivity (1 μg/mL), intra-assay variation (<10%) and inter-assay variation (CV < 15%).


$$\begin{array}{c} \mathrm{OD}\;\mathrm{value of}\;\mathrm{IgM}=\mathrm{OD}\;\mathrm{value of measuring tube}\\ -\mathrm{OD}\;\mathrm{value of control tube} \end{array}$$

The concentration of IgM in plasma (μg/mL) was also a measure of the linear regression equation of the standard curve by using the standard concentration and OD value, substituting the OD value of the sample into the equation of the standard curve, which estimated the sample concentration. Finally, it was multiplied by the dilution factor for the actual concentration of IgM in the sample.

### 2.7. Statistical analysis

Data were analyzed using statistical software (GraphPad Prism-6 Software, San Diego, CA, United States). The observed variables in the current study were initially observed for normality through Shapiro–Wilk test, and observed data were expressed as means ± SEM. The One-way analysis of variance (ANOVA) was used to compare the reproductive variables (follicular dynamics, duration to estrus or ovulation) among groups. Data of pregnancy, estrus and ovulation across the groups were compared through the chi-square test using the Prism-6 software package (GraphPad Software, San Diego, CA, United States). An effect of time (days after melatonin injections) and treatment (single or multiple melatonin injections) on serum melatonin or IgM level and milk traits was observed using ANOVA with repeated measures. The comparison of means was analyzed by using SPSS Statistics for Windows, version 23.0 (IBM Corp. Armonk, NY, United States). The difference between the groups was declared statistically significant if *p*-value observed <0.05.

## 3. Results

**Figure 2:**
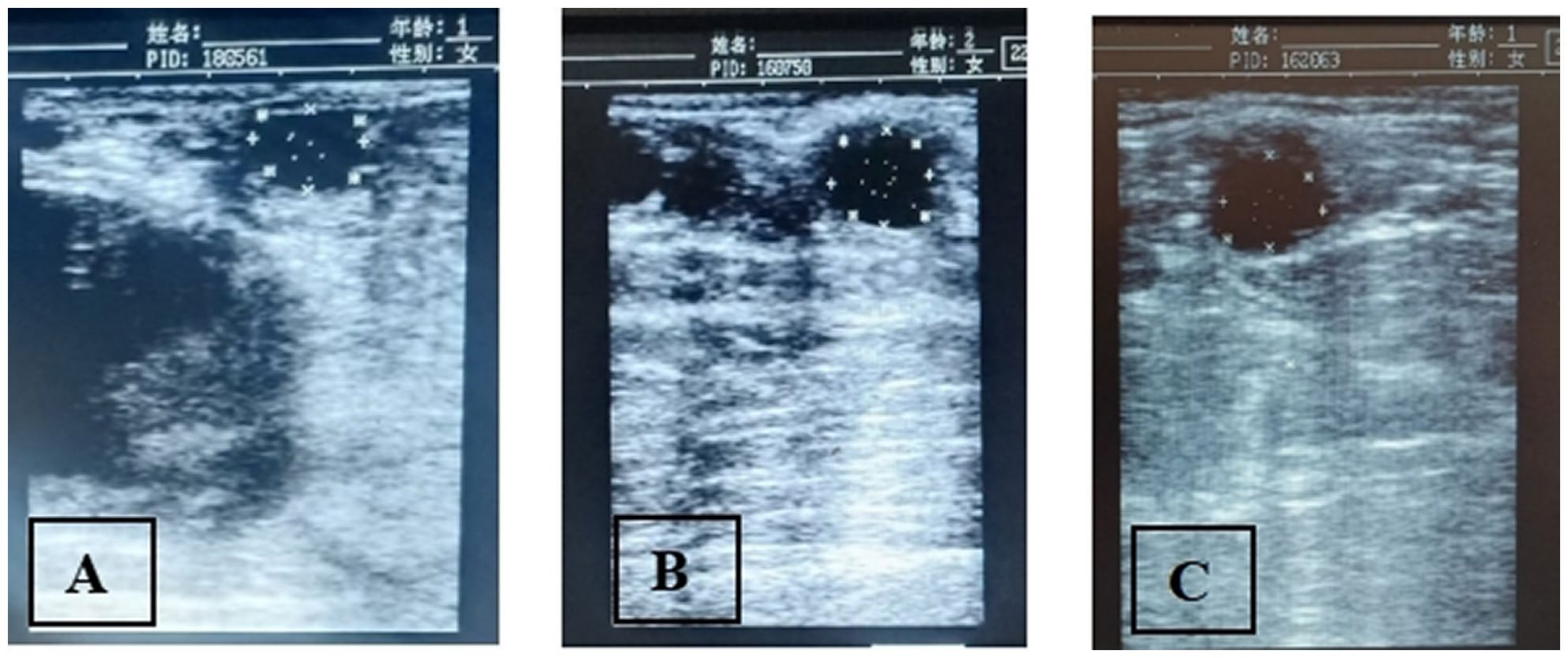
The sonograms showing the ovulatory follicle diameter in melatonin treated [(**A**; G-1), (**B**; G-2)] and control (**C**; G-3) groups of crossbred buffaloes synchronized through modified Ovsynch.

**Figure 3:**
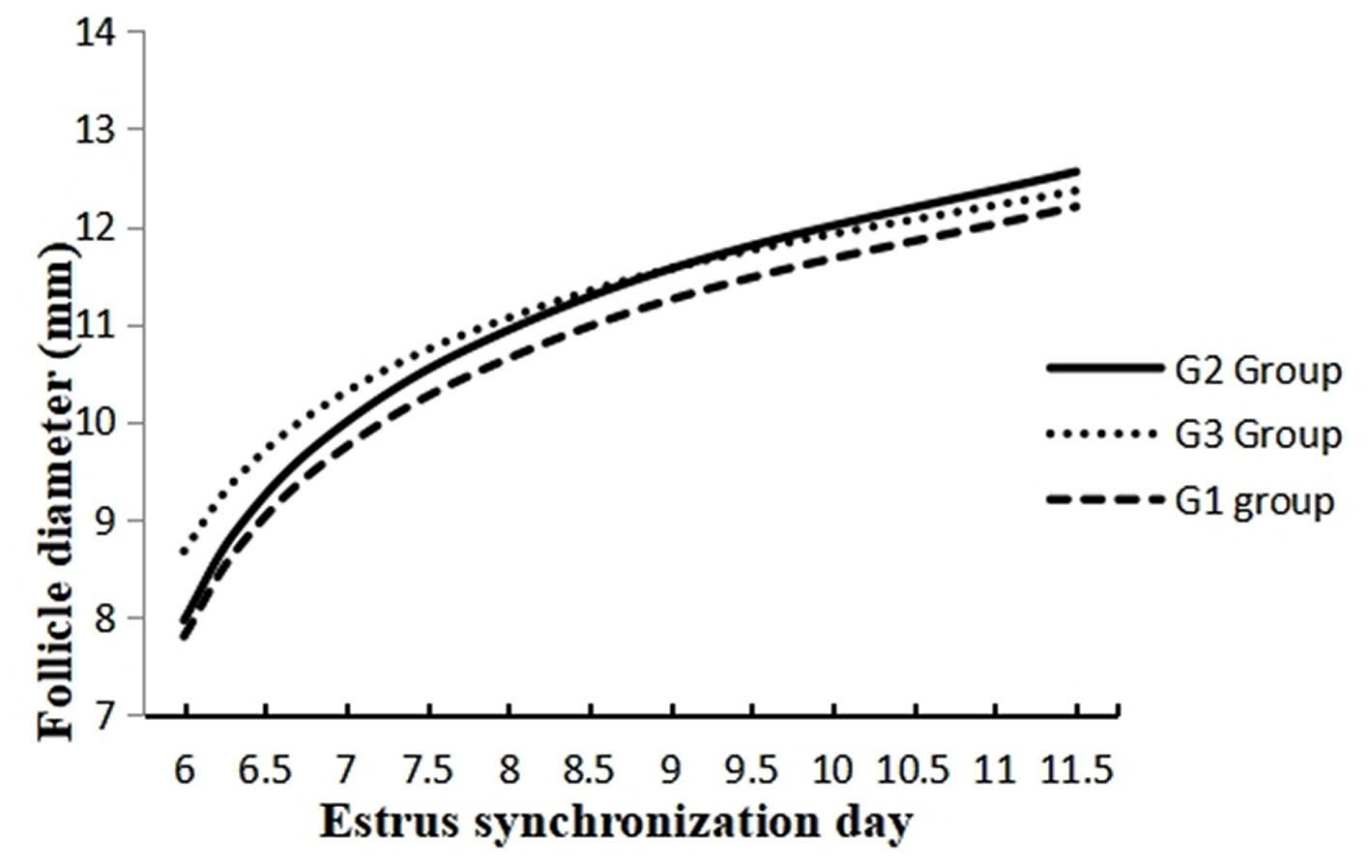
Follicle development at different days of estrus synchronization in melatonin treated (G1 and G2) and control crossbred buffaloes.

The data on the effect of melatonin on follicular dynamics, estrus behavior, ovulation and conception rates are presented in Table 1. The results revealed that the studied groups had similar follicle diameter on day 6, the interval between 2nd GnRH injection and estrus duration. Estrus response and ovulation rates were significantly higher (*p* < 0.05) in the G2 than in the G1 group. There was no difference in interval between 2nd GnRH and ovulation, ovulatory follicle size (Figure 2), and follicular cysts incidence among treatment groups. The follicular growth rate was significantly greater (*p* < 0.05) in G2 and G3 groups as compared G1 group (Figure 3). Similarly, the pregnancy rate was also significantly higher (*p* < 0.05) in G2 and G3 groups than in the G1.

**Table 1:** Effect of melatonin before initiation of modified Ovsynch protocol on follicle dynamics, estrus, ovulation and pregnancy rate in crossbred buffaloes during low breeding season.

Parameters	Treatment groups		
	G1 (*n* = 20)	G2 (*n* = 22)	G3 (*n* = 23)
Follicle diameter at day 6 of protocol (mm)	8.2 ± 1.7	8.7 ± 1.7	8.9 ± 1.9
Estrus rate (%)	11/20 (55)^b^	19/22 (86.4)^a^	17/23 (73.9)^ab^
Interval between 2nd GnRH and estrus (h)	9.5 ± 1.2	8.7 ± 1.7	9.1 ± 1.1
Estrus duration (h)	16.4 ± 1.5	15.7 ± 0.7	16.0 ± 1.3
Ovulation rate (%)	10/20 (50.0)^b^	18/22 (81.8)^a^	16/23 (69.6)^ab^
Interval between 2nd GnRH and ovulation (h)	24.3 ± 0.9	23.6 ± 1.1	24.5 ± 1.5
Ovulatory follicle size (mm)	14.0 ± 1.6	15.7 ± 1.9	14.7 ± 1.9
Follicle growth (mm/day)	0.7 ± 0.18^b^	1.8 ± 0.32^a^	1.5 ± 0.15^a^
Follicular cysts incidence (%)	2/20 (10)	0/22 (0)	1/23 (4.3)
Pregnancy rate (%)	5/20 (25.0)^b^	12/22 (54.5)^a^	10/23 (43.5)^a^

Results are presented as Mean ± SEM.

Different superscripts ^(a,b)^ represent significant differences (*P* < 0.05) within the same row.

G1, Control group; G2, Single injection of melatonin; G3, multiple injection of melatonin.

Data on serum concentrations of melatonin and IgM are depicted in Table 2. The serum concentration of melatonin has risen significantly (*p* < 0.05) in both G2 and G3 groups on day 1 compared to other days. In contrast, serum melatonin concentration was not statistically different across the groups at the time of melatonin administration, day 5 or 10 of observation, respectively. However, there was a significantly higher melatonin concentration in both melatonin-treated groups (G2 and G3) on day 1 after administration than in the untreated one.

**Table 2:** Blood profile of melatonin and IgM levels at different times following exogenous melatonin treatment in crossbred buffaloes during summer season.

Variables	Groups	Post-melatonin treatment (days)			
		0	1	5	10
Melatonin level (pg/mL)	G1 (*n* = 20)	192.4 ± 12.6	195.31 ± 13.8^B^	192.58 ± 17.5	194.85 ± 18.1
	G2 (*n* = 22)	198.79 ± 31.52^b^	528.60 ± 58.21^Aa^	239.50 ± 38.12^b^	249.50 ± 22.12^b^
	G3 (*n* = 23)	191.57 ± 27.83^b^	382.81 ± 33.72^ABa^	197.21 ± 19.46^b^	187.21 ± 15.36^b^
IgM (μg/L)	G1 (*n* = 20)	268.19 ± 41.28	277.0 ± 42.0^B^	266.87 ± 42.56^B^	275.88 ± 41.96
	G2 (*n* = 22)	318.79 ± 48.52^b^	521.60 ± 58.21^Aa^	409.50 ± 38.12^Ab^	329.50 ± 45.16^b^
	G3 (*n* = 23)	211.57 ± 47.83^b^	412.81 ± 33.72^Aa^	287.21 ± 39.46^Bb^	307.21 ± 29.46^b^

Results are presented as Mean ± SEM.

Small superscripts ^(a,b)^ along rows and large superscripts ^(A,B)^ along column indicate the significant difference (*P* < 0.05).

G1, Control group; G2, Single injection of melatonin; G3, multiple injection of melatonin.

The serum IgM level has increased significantly (*p* < 0.05) in the G2 and G3 groups compared to the G1 group after 1 day of melatonin administration. The serum IgM level was also statistically (*p* < 0.05) higher in the G2 than G1 and G2 groups after 5 days of melatonin administration.

The effect of melatonin administration on milk production and composition is illustrated in Table 3. Data on the milk yield (liters) and composition traits (fat, lactose, non-fat solids, and total solids) exhibited a statistically non-significant difference among the treatment groups. Milk protein was significantly reduced in the G1 group compared to the G2 and G3 groups at both observational times after melatonin administration. Similarly, milk urea nitrogen was significantly lower in the G3 than in the G1 group after 10 days of melatonin administration. However, the somatic cell count was significantly lower in the G3 and G2 groups as compared G1 group after 10 days of melatonin administration.

**Table 3:** Effect of exogenous melatonin treatment on milk production (liters/head) and composition traits (fat, protein, lactose, non-fat solids, total solids, milk urea nitrogen and somatic cell count) in buffaloes.

Milk traits	After melatonin injection (days)	G1 (*n* = 20)	G2 (*n* = 22)	G3 (*n* = 23)
Milk yield (liters/head)	5	3.23 ± 1.6	2.98 ± 1.8	3.04 ± 1.9
	10	4.48 ± 2.9	3.50 ± 2.5	4.11 ± 2.9
Milk fat (%)	5	7.33 ± 1.4	7.71 ± 1.2	7.85 ± 1.8
	10	7.52 ± 1.3	7.89 ± 1.4	7.90 ± 1.4
Milk protein (%)	5	4.17 ± 0.5^b^	4.39 ± 0.5^Ba^	4.37 ± 0.5^a^
	10	4.22 ± 0.4^b^	4.59 ± 0.5^Aa^	4.39 ± 0.6^a^
Lactose (%)	5	4.78 ± 0.6	4.93 ± 0.6	5.0 ± 0.7
	10	4.87 ± 0.5	5.04 ± 0.5	5.13 ± 0.5
Non-fat solids (%)	5	10.08 ± 0.5	10.12 ± 0.7	10.2 ± 0.8
	10	10.1 ± 0.5	10.37 ± 0.8	10.51 ± 0.8
Total solids (%)	5	17.82 ± 1.2	17.86 ± 1.9	18.12 ± 1.7
	10	17.83 ± 1.7	18.09 ± 1.9	18.59 ± 2.6
Milk urea nitrogen (mg/dL)	5	12.74 ± 3.2	13.54 ± 1.0	13.49 ± 3.7
	10	11.92 ± 1.8^b^	13.69 ± 3.9^ab^	14.11 ± 2.1^a^
Somatic cell count (10^3^/mL)	5	62.67 ± 22.54	60.26 ± 18.9	63.72 ± 24.7
	10	63.09 ± 16.5^a^	47.72 ± 6.2^b^	46.12 ± 20.3^b^

Different superscripts ^(a,b)^ represent significant differences (*P* < 0.05) across groups in same row, while ^(A,B)^ represent significant differences (*p* < 0.05) within the group in same column.

Results are presented as Mean ± SEM.

G1, Control group; G2, Single injection of melatonin; G3, Multiple injection of melatonin.

## 4. Discussion

The present study demonstrated that melatonin administration, in injectable form, influences follicular dynamics, estrus, ovulation, and pregnancy rates in crossbred buffaloes during the low breeding season (Table 1). The first GnRH hormone injection was administered at day 0 (1 day after melatonin administration) of the modified Ovsynch protocol that results in ovulation of the dominant follicle (35, 36). The melatonin administration did not affect the follicular diameter of the first largest follicle on day 6 of the protocol following the first GnRH injection. In the present study, melatonin usage has improved the estrus rate in crossbred buffaloes. Usually, buffaloes express their estrus signs in the late evening and early morning (37), but no or weak estrus signs were observed during summer in tropical and sub-tropical countries (1, 38). It is observed that melatonin administration along various synchronization regimens enhanced estrus expression in different species (39, 40) and increased uterine blood flow with estrus induction (41). On the contrary, Kavita et al. (42) reported no effect on the estrus response of treated animals. The obtained results of ovulation rate also indicated a positive relationship with melatonin administration. This observation aligns with claims reported in sheep (43, 44) and buffaloes (45). On the contrary, Sweeney and O’Callaghan (46) did not find any improvement in ovulation rate following melatonin administration. The influence of melatonin administration on intervals between 2^nd^ GnRH administration and ovulation in the current study corresponds with previous observations in buffaloes (45). The previous studies indicated an increasing trend of ovulatory follicles in heifers and lactating buffaloes after melatonin treatment along CIDR application (18, 19) or Ovsynch protocol (45). A similar pattern of ovulatory follicles was observed in the present study when crossbred buffaloes were synchronized with modified Ovsynch protocol. In an earlier report, a melatonin implant (18 mg/50 kg BW) successfully induced estrus in anestrous heifers and multiparous buffaloes (27, 47). In the case of goats, the use of melatonin injection improved the pregnancy outcomes during the out-breeding season (47, 48) and melatonin use prior to the breeding season also reduced the anestrous period and initiated the cyclicity in goats earlier (48). In this context, using melatonin in anestrous buffaloes in the late low breeding season could be an ameliorative strategy to reduce the duration of the anestrous period.

The current study observed a positive association between melatonin administration and pregnancy rate. Single and multiple melatonin administration has significantly improved pregnancy outcomes in crossbred buffaloes during the low breeding season. The pregnancy rate results agreed with the earlier reports (16, 17, 42) when buffaloes were treated with melatonin, in injectable or slow-release forms, during the low breeding season. Previous reports proved that melatonin administration improved fertility and fecundity in ewes (49–51). A higher percentage of pregnancy rates might be associated with an improvement in fertilization which is connected to the antioxidant capacity of melatonin (52). The mechanisms of improving embryo viability in the present study by melatonin administration might be linked to neuroendocrine stimulation and the antioxidant effect of melatonin that protects the oocyte from oxidative stress (53) and enhances oocyte maturation and cleavage rate (54).

In the present study, using melatonin in injectable form rapidly increased the peripheral circulation level and subsequent IgM values. This abrupt increase in melatonin levels in blood circulation is similar to earlier reports where melatonin implants were used (55, 56). The serum melatonin levels post-melatonin treatment were comparatively higher than the previous reports (16, 42), which might be associated with the sensitivity of the used assay or sampling time. In the present study, blood samples were collected during dark hours, which might trigger the endogenous melatonin secretion that increased the serum melatonin level compared to earlier reports. Administering melatonin injection through the subcutaneous route is more manageable than implant placement subcutaneously. It is documented in cows that using melatonin through intravenous or subcutaneous route improves melatonin concentration rapidly with the improved metabolic rate (57, 58). However, a detailed comparison of melatonin administration routes on melatonin level, hormonal response, oxidative changes, metabolic response and fertility results is needed in buffaloes.

Melatonin treatment reduces milk yield in cows (59). In the current study, melatonin treatment did not influence milk volume. Similar results were reported by Cosso et al. (60) that melatonin does not affect milk volume in sheep. The data indicated that melatonin does not affect milk fat, whereas recent reports claimed it reduced milk fat after melatonin treatment (61, 62). In contrast, the increased milk fat in cows was reported by Auldist et al. (59) that improvement might be linked to the modulatory effect of melatonin on milk fat content by suppressing milk fat synthesis through inhibition of the mTOR signaling pathway *via* the MT1 receptor in BMEC (63). In the current study, milk protein decreased after 5 days of melatonin treatment, but others milk parameters like lactose and total solids remained unaffected. Contrary to the current study, melatonin has increased lactose and protein levels (60, 61). Similarly, milk protein and casein increased in a previous study with reduced lactose (58). The findings of Cosso et al. (59) also follow our results which indicate no effect of melatonin on milk yield or milk composition in sheep. In contrast, melatonin positively affects milk yield, protein, lactose, fatty acids and fat in sheep (64).

The number of somatic cells in milk is a very important indirect indicator to measure the health status of cow udders (32). However, melatonin can regulate immune function by regulating cytokine production (65) and can improve the body’s antioxidant level (66). In the current study, milk somatic cell count was reduced following different subcutaneous melatonin injections after 10 days of administration. Similar observations were documented in normal Holstein cows (27), dairy goats (67), and mastitis-affected cows (60, 61). The increased SCC due to environmental stress (heat and cold) can be controlled by improving immunity through melatonin administration (26). It is postulated that melatonin can be beneficial in different infections due to its anti-inflammatory and anti-oxidative activities. Melatonin reduces oxidative stress and inhibits inflammatory cytokines, regulating the activation of STATs and NF to alleviate bovine mastitis (68).

## 5. Conclusion

Taken together, the current data suggest that multiple doses of melatonin prior to the application of the modified Ovsynch protocol improved the reproductive performance of crossbred buffaloes during the low breeding season. Moreover, the administration of melatonin enhanced the IgM values along milk traits in terms of milk protein, MUN and somatic cell count in treated buffaloes.

## Data availability statement

The original contributions presented in the study are included in the article/Supplementary material, further inquiries can be directed to the corresponding authors.

## Ethics statement

The animal study was reviewed and approved by the Animal Welfare and Ethical Committee, Huazhong Agriculture University, People’s Republic of China (Approval ID: HZAUBU-2017-001). Written consent was obtained from the owners for the use of animals in this study.

## Author contributions

AA, WL, MA, AfS, XP, AsH, and SW designed and conducted the experiment. AA, MN, ZN, AfS, ZA, AsH, and WL wrote and revised the manuscript. AA and SW analyzed the data. XP and SW supervised the whole experiments. All authors have reviewed the manuscript and agreed to publish this version as manuscript.

## Funding

This study was financially supported by the Natural Science Foundation of Anhui Province (2008085MC94), the National Natural Science Foundation (31301972), Key Research and Development Project of Chuzhou (2018ZN014), Key Research and Development Program of Anhui Province (202004f06020048), and Science and Technology Project for Enhancing Competitiveness Industry of Cattle and Sheep in Anhui Province (AHCYJSTX-07-202111).

## Conflict of interest

The authors declare that the research was conducted in the absence of any commercial or financial relationships that could be construed as a potential conflict of interest.

## Publisher’s note

All claims expressed in this article are solely those of the authors and do not necessarily represent those of their affiliated organizations, or those of the publisher, the editors and the reviewers. Any product that may be evaluated in this article, or claim that may be made by its manufacturer, is not guaranteed or endorsed by the publisher.
